# Evaluation of the Stability of Internal Fixation Constructs in a Low Distal Tibia Extra-articular Cadaveric Fracture Model: A Comparative Biomechanical Study

**DOI:** 10.7759/cureus.54033

**Published:** 2024-02-11

**Authors:** Tarkik Thami, Siddhartha Sharma, Amit Kumar, Naveen Kumar, Nitin Chauhan, Anjali Aggarwal, Mandeep Dhillon

**Affiliations:** 1 Orthopaedics, Postgraduate Institute of Medical Education and Research, Chandigarh, Chandigarh, IND; 2 Mechanical Engineering, Indian Institute of Technology (IIT), Ropar, IND; 3 Orthopaedics, All India Institute of Medical Sciences, New Delhi, New Delhi, IND; 4 Anatomy, Postgraduate Institute of Medical Education and Research, Chandigarh, Chandigarh, IND

**Keywords:** locking compression plate, intramedullary nailing, biomechanical study, extra-articular fractures, distal tibia

## Abstract

Introduction

Despite the recent advances in implant design, the choice of an internal fixation modality for extra-articular distal tibia fractures remains controversial, and there is sparse literature comparing the stability of intramedullary nails and locked plates for such fractures. Hence, we conducted a biomechanical study on an AO type 43A3 tibia fracture cadaveric model stabilized by four different constructs, viz., intramedullary (IM) interlocking nail, anteromedial plate, anterolateral plate, and posterior plate. An AO type 43A3 fracture is defined as an extra-articular fracture of the distal tibia with metaphyseal comminution.

Methods

A biomechanical comparative study on formalin-preserved human cadaveric tibiae was undertaken; a total of four groups were tested, with eight bones in each group. Out of the 32 cadaveric tibiae, 19 bones belonged to male cadavers, and 13 bones belonged to female cadavers. All bones were dissected from age-appropriate cadavers and fixed with an implant, followed by the creation of a 1 cm osteotomy to simulate an AO type 43A3 fracture. All fixation constructs were subjected to three-point bending tests in the anteroposterior (AP) and mediolateral (ML) planes. Three parameters, viz., bending stiffness, peak fracture gap angle, and neutral zone, were evaluated on the load-displacement curves. A fixation construct was deemed biomechanically stable if it had a high bending stiffness, a low neutral zone (inherent toggle in the construct by its weight), and a low peak fracture gap angle.

Results

Out of the four implants tested, locked IM nails exhibited the maximum biomechanical stability in terms of higher bending stiffness, smaller peak fracture gap angle, and smaller neutral zones. The IM nail exhibited the highest bending stiffness in the AP plane, and the anterolateral plate had the lowest bending stiffness, and the difference was statistically significant (p= 0.032). In the AP plane, the anterolateral plate exhibited a bending stiffness of 1.51 ± 0.69 Nm/degree, whereas the intramedullary nail exhibited a bending stiffness of 2.34 ± 0.81 Nm/degree, and the posterior locked plate had a bending stiffness of 1.57 ± 0.44 Nm/degree. In the ML plane, the anterolateral plate exhibited the highest neutral zone as compared to the intramedullary nail, which had the lowest neutral zone, and the difference was statistically significant (p = 0.019). The intramedullary nail exhibited the lowest neutral zone of 0.46 ± 0.31 degrees, whereas the posterior locked plate exhibited a neutral zone of 0.78 ± 0.43 degrees in the ML plane. The anterolateral plate exhibited a neutral zone of 1.43 ± 1.00 (expressed as mean ± SD) degrees in the mediolateral plane.

Conclusion

Our biomechanical study supports the recommendations of using a locked intramedullary nail for AO type 43A3 fractures. We concluded that the anterolateral plate construct exhibited the least biomechanical stability, in terms of lower AP bending stiffness and higher neutral zone. If the surgeon must choose a locked plating technique for any reason, the anterolateral locking plate should be avoided. If plating is at all required, we can recommend both anteromedial and posterior locked plating as biomechanically sound options.

## Introduction

Distal tibial fractures are complex injuries that present with a unique set of problems, owing to the subcutaneous nature of the bone, relatively poor blood supply, and proximity to the ankle joint [1,2]. Low extra-articular distal tibial fractures are a special subgroup where the fracture line is close to the tibial plafond, which results in a very short distal fragment [3]. Conventional intramedullary (IM) nailing in this subgroup of fractures is often biomechanically inadequate because of the short length of the distal fragment, which could result in residual instability in the coronal or sagittal plane [4-6]. Extramedullary implants like locked plates also need to be used cautiously because of complications like implant prominence and wound dehiscence [7,8]. The current literature on the comparison of the stability of different osteosynthesis modalities for low, extra-articular distal tibia fractures is sparse [9,10]. Hence, this study was conducted to compare the biomechanical stability of four different internal fixation constructs in a low, unstable, distal tibia, extra-articular fracture model (AO type 43A3). We hypothesized that there would be no difference in the biomechanical stability of these four internal fixation constructs.

## Materials and methods

Study design

This was a biomechanical study conducted on formalin-preserved human cadaveric tibiae. The study was approved by the Institutional Ethics Committee, PGIMER, Chandigarh (INT/IEC/2020/SPL-319).

Sample size and study groups

This study consisted of a total of four experimental groups, representing low, extra-articular distal tibia fractures stabilized by four different internal fixation constructs. Cadaveric tibiae were measured with the help of measuring tape, and the range of length was 34-38 cm. Out of 32 cadaveric tibiae, 19 bones belonged to male bodies, and 13 bones belonged to female bodies.

Group A: Stabilization of the fracture by an anterolateral locking plate construct. Commercially available distal tibial anterolateral locking plates were used (Siiora Surgicals, Sonipat, India). Four bicortical locking head screws (3.5 mm) were placed in the distal fragment, and four bicortical locking screws (3.5 mm) were placed in the proximal fragment.

Group B: Stabilization of the fracture by an anteromedial locking plate construct. Commercially available distal tibia anteromedial locking plates were used (Siiora Surgicals, Sonipat, India). Four bicortical locking head screws (3.5 mm) were placed in the distal fragment, and four bicortical locking screws (3.5 mm) were placed in the proximal fragment.

Group C: Stabilization of the fracture by an intramedullary interlocking nail construct. Commercially available interlocking nails specifically designed for the distal tibia were used for the study (Siiora Surgicals, Sonipat, India). A reamed nailing technique was followed, and the nail was locked proximally (two proximal bolts, both static and dynamic) and distally with three interlocking bolts: two mediolateral and one anteroposterior. All interlocking bolts had a bicortical purchase.

Group D: Stabilization of the fracture by a posterior locking plate construct. The commercially available anterolateral distal tibia anatomical plates were used (Siiora Surgicals, Sonipat, India) but were applied posteriorly. We added this group to our study as there was limited literature on the biomechanical testing of this fixation construct [11]. Four bicortical locking head screws (3.5 mm) were placed in the distal fragment, and four bicortical locking screws (3.5 mm) were placed in the proximal fragment.

Tibia fracture creation

**Figure 1:**
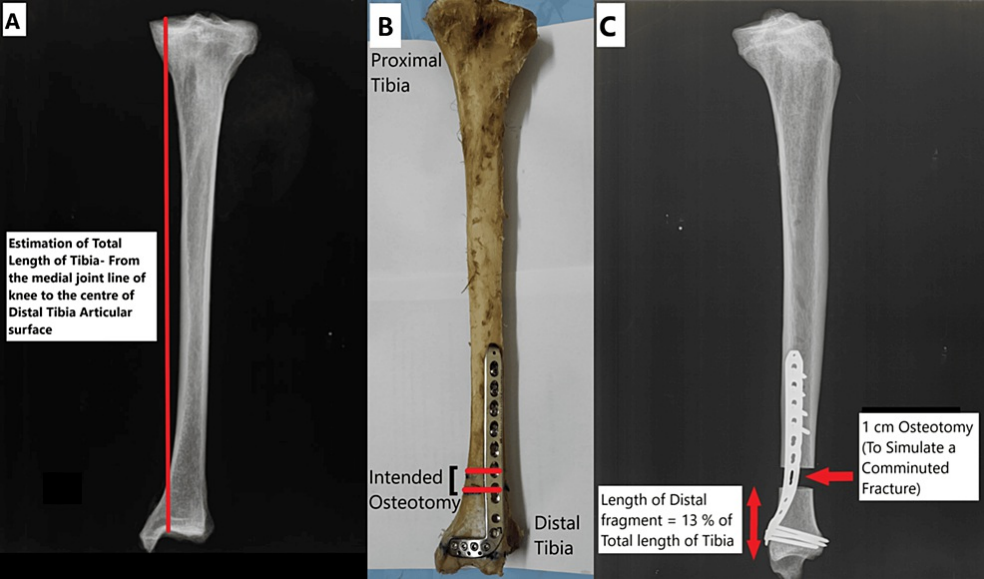
(A) Method of estimation of the total length of tibia. (B) Depiction of the posterior aspect of the tibia with the marked site of intended osteotomy. (C) Post-osteotomy radiograph demonstrating the length of the distal fragment.

An AO type 43A3 fracture pattern was created after the insertion of the internal fixation implants. A 1 cm transverse gap (osteotomy) was created at the intended site using an oscillating saw, taking care not to damage the implant. To standardize for variations in tibial length, the osteotomy was performed to create a distal fragment that was 13% of the total tibial length (measured from the highest point of the anterior medial tibial plateau rim to the center of the anterior rim of the tibial plafond) [12] (Figures 1A, 1B). For example, the osteotomy site for a tibia measuring 40 cm was placed 5.2 cm (40 x 0.13 = 5.2) above the tibial plafond. The osteotomy pattern was intended to simulate a low, comminuted, extra-articular distal tibial fracture (Figures 1B, 1C) with no contact between the proximal and distal fracture segments and no support by the fibula or the interosseous membrane. This particular fracture pattern represents a highly unstable distal tibial fracture pattern.

Radiological assessment

Each formalin-preserved human cadaveric tibia was subjected to radiographs (standard orthogonal AP and lateral views) both before fixation (Figure 1A) and after the creation of an osteotomy (Figure 1C).

Biomechanical testing

**Figure 2:**
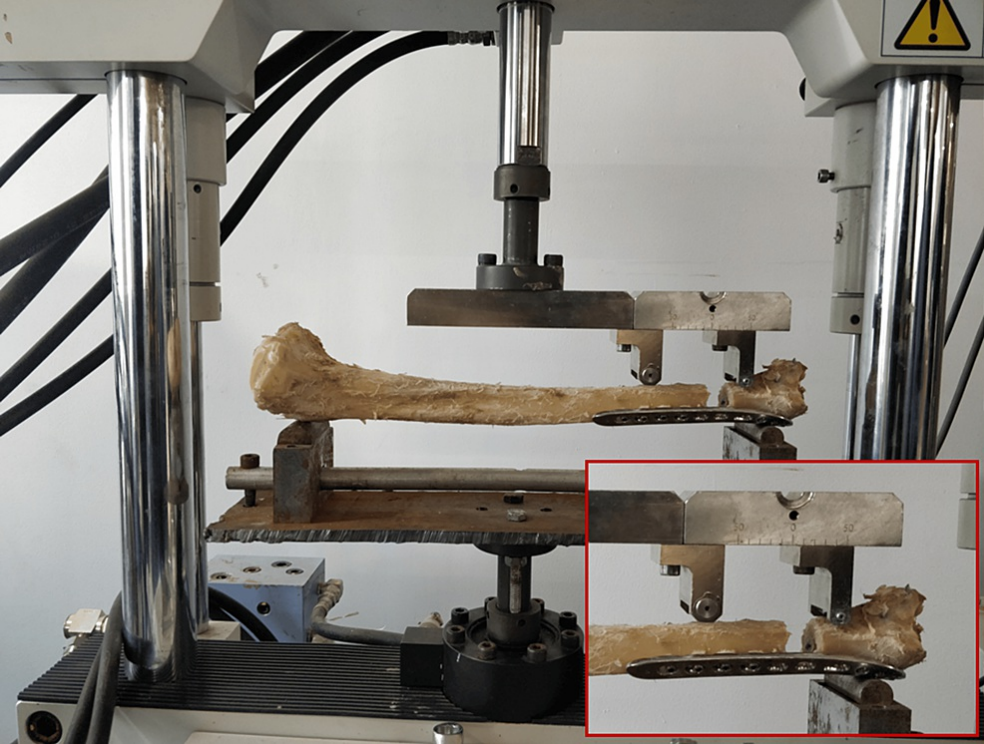
Three-point bending performed on a servo-hydraulic biomechanical testing machine.

All fixation constructs were subjected to three-point bending tests in the anteroposterior (AP) and mediolateral (ML) planes. Biomechanical testing was conducted using a servo-hydraulic testing machine (Shimadzu Servopulser EHF-E series fatigue testing machine; Shimadzu Corporation, Kyoto, Japan) (Figure 2). One bone in each group was used for pilot testing and standardization [10]. A specific preload was applied to the bone, and the elastic range of the test specimen was calculated. Three-point bending tests were undertaken on a customized jig, comprising two adjustable support rollers. A load of approximately 150 N; equivalent to 15.6 kg, was applied with an actuator speed of 0.05 mm/sec (within the elastic limit) on the implanted tibia, followed by unloading at a reduced speed of 0.0025 mm/sec. The load and displacement curves were recorded, and the mean slope was calculated between 50 and 150 N (linear elastic region) [9,10,12].

Outcome measures

The bending stiffness was evaluated in the mediolateral (coronal) and anteroposterior (sagittal) planes and was calculated by the slope of the load-displacement curve [10,13]. Bending stiffness was expressed as Nm/degree.

The peak fracture gap angle was defined as the maximum displacement (in degrees) noted during three-point bending.

**Figure 3:**
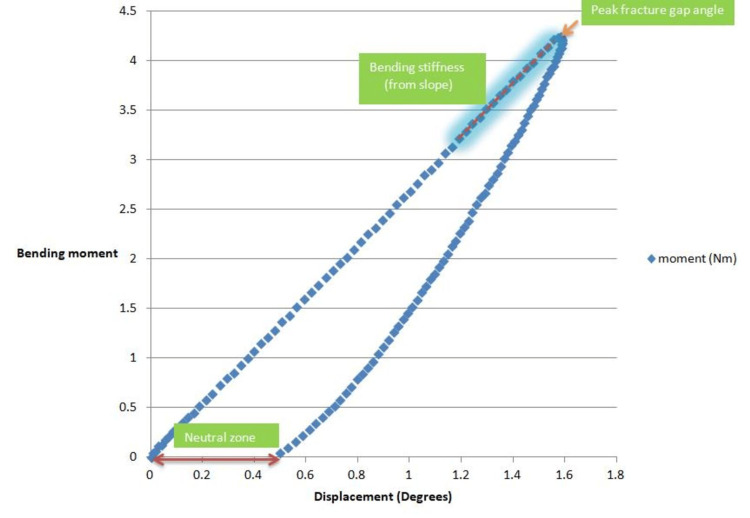
A load-displacement curve obtained from three-point bending, depicting the key outcome parameters evaluated in the study.

The neutral zone was defined as the difference in displacement between the loading and unloading regions of the load-displacement curve. The neutral zone represents the inherent degree of instability in a construct. The lower the neutral zone, the more stable the construct (Figure 3).

After an appropriate analysis of results, a construct was deemed more stable if it had a lower peak fracture gap angle, lower neutral zone, and higher bending stiffness.

## Results

**Figure 4:**
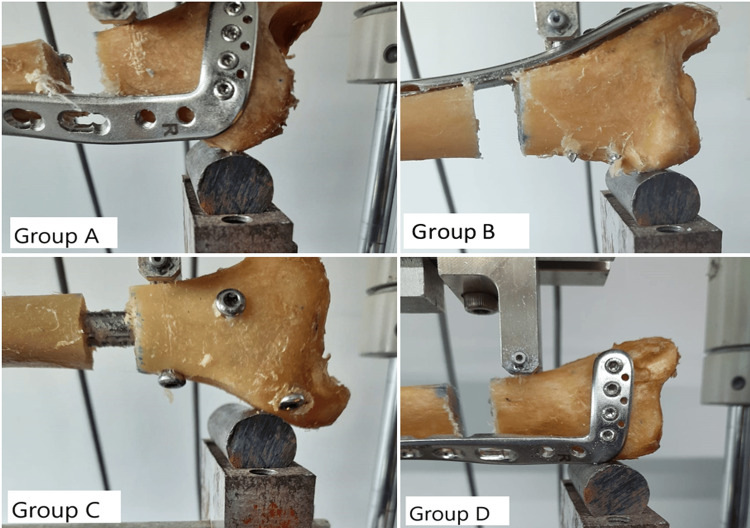
Depiction of three-point bending tests for fixation constructs of Groups A, B, C, and D.

All study groups underwent biomechanical testing (three-point bending) (Figure 4) after preload testing of one bone in each group. Fixation-construct failure was not found in any bone.

Mediolateral three-point bending

**Figure 5:**
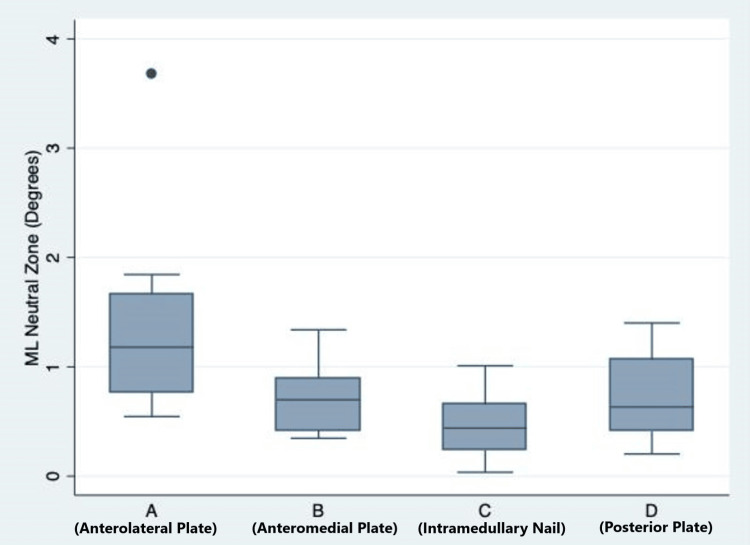
Box and whisker plot, showing a comparison of the mediolateral neutral zone among the four study groups. The solid line in the middle of the box represents the median value, and the upper and lower limits of the box represent the interquartile range.

The mean ML bending stiffness was lowest for Group A (anterolateral plating) and highest for Group C (intramedullary nailing), but a one-way ANOVA did not reveal a statistically significant difference across the four groups (p = 0.0581). The mean peak fracture gap angle (mediolateral) was highest for Group A and lowest for Group C, but the difference was not statistically significant across the four groups (p = 0.335). Group A had a higher neutral zone than Group C (in the mediolateral plane), and the difference between groups was statistically significant (p = 0.019) (Figure 5).

Anteroposterior three-point bending

**Figure 6:**
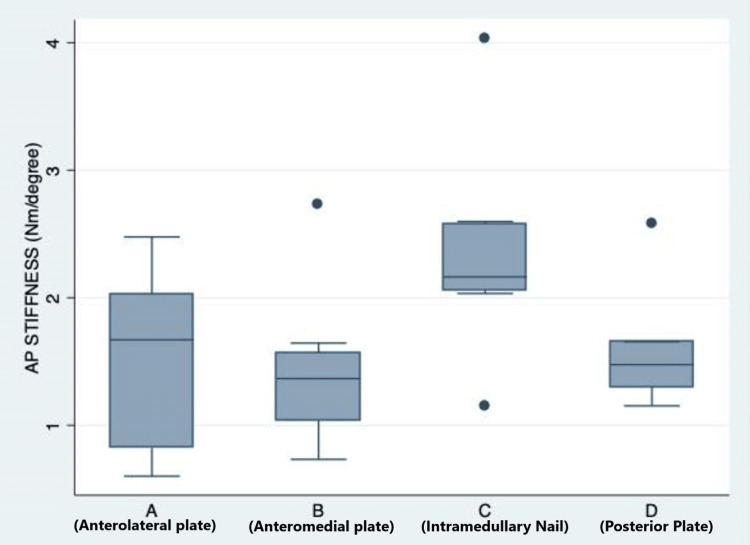
Comparison of anteroposterior bending stiffness (Nm/degree) among the four study groups. The solid line in the middle of the box represents the median value, and the upper and lower limits of the box represent interquartile range.

There was a significant difference in anteroposterior bending stiffness across the four groups (Figure 6). A post hoc analysis (using Tukey's honest significance test (HSD)) revealed a significant difference between Groups A and C (p = 0.032) (higher bending stiffness in Group C as compared to Group A).

The average peak fracture gap angle was highest for Group A (anterolateral plating) and lowest for Group C (intramedullary nailing), but a one-way ANOVA did not reveal a statistically significant difference across the four groups (p = 0.204). The average neutral zone was highest for Group B (medial plating) and lowest for Group D (posterior plating), but a one-way ANOVA did not reveal a statistically significant difference across the four groups (p = 0.087).

Summary of results

The intramedullary interlocking nail exhibited maximum biomechanical stability, in terms of higher anteroposterior and mediolateral bending stiffness, smaller peak fracture gap angle, and smaller neutral zone, as compared to the other three constructs. Out of the three plates tested, the anterolateral locked plate had the least biomechanical stability, in terms of lower anteroposterior and mediolateral bending stiffness, higher peak fracture gap angle, and higher neutral zone. A statistically significant difference was noted in the anteroposterior bending stiffness of the anterolateral plate versus the intramedullary nail (lower with plate) and mediolateral neutral zone of the anterolateral plate versus intramedullary nail (higher with plate).

## Discussion

Distal tibia fractures are quite challenging to treat, owing to the thin, soft tissue cover available in this region and the precarious blood supply [7]. The choice of operative fixation can be influenced by various factors, which include soft tissue condition, type and pattern of the fracture, degree of comminution, bone quality, and length of the distal fragment available for a stable osteosynthesis [6,12-15]. Intramedullary nailing for distal tibia fractures is reported to have the benefits of shorter operative times, the ability to be used in a minimally invasive fashion, and the preservation of fracture biology [16,17]. Conversely, anatomically contoured distal tibia plates provide the surgeon with the option of multiple, angle-stable (locking) screws in the short distal fragment, and they are reported to have lower malunion rates [6].

Although the debate on radiological and functional outcomes of intramedullary nails versus anatomical locked plates (for extra-articular distal tibia fractures) continues, a thorough literature review reveals that there is sparse literature comparing the biomechanical stability of different osteosynthesis modalities for these injuries [18]. Based on the results of our study, we noted that the anterolateral plate (Group A) had the lowest biomechanical stability in terms of lower anteroposterior bending stiffness and higher neutral zone.

The “neutral zone” refers to the amount of “play” or “toggle” within a construct. It is the amount of movement permitted in the construct by its weight, and it reflects the inherent instability of the fracture-implant construct; the higher the neutral zone the more the instability [19]. However, it should be noted that as fracture healing progresses, this instability will potentially decrease. In our study, the neutral zone was the lowest for locked intramedullary nails. This may be explained by the intramedullary nature of the implant and the presence of locking bolts, which further minimize any toggle of the distal fragment. The anterolateral locking plate, on the other hand, had the highest neutral zone; this may be attributed to the plate design. This L-shaped plate has a distal, horizontal limb that rests on the anterior part of the tibial plafond and a proximal, vertical limb that rests on the anterolateral surface of the tibial shaft. As the plate position on the tibial shaft is neither strictly anterior nor lateral, a significant toggle can be expected in both the anteroposterior and mediolateral planes.

The “bending stiffness” refers to the resistance against deforming forces; the higher the stiffness, the more stable the construct. We noted that the locked intramedullary nail had the highest bending stiffness in the anteroposterior and mediolateral planes [13]. On the other hand, the anterolateral plate had the lowest bending stiffness in both planes. The posterior locked plating construct was noted to have a marginally higher bending stiffness than the anteromedial locked plate construct, although this result was not statistically significant. Hence, our biomechanical testing supports the recommendations of using a locked intramedullary nail for low distal tibia extra-articular fractures. The load-sharing nature of the nail imparts higher biomechanical stability, and its minimally invasive nature allows for the preservation of biology [10]. Moreover, problems because of soft tissue irritation that often occurs with the anterolateral or anteromedial locked plates can be avoided with the intramedullary nail [7].

If the surgeon must use a locked plate for extra-articular distal tibial fractures, we can recommend both the anteromedial and posterior locked plating because the results of our study support the biomechanics of the posterior locked plate followed by the anteromedial locked plate construct. In select instances where the length of the distal fragment is insufficient to accommodate a minimum of three locking bolts of an intramedullary nail, the surgeon does not have an option but to resort to plating. Based on our results, we can say that the anterolateral plating construct has the least biomechanical stability when compared to the anteromedial and posterior locking plate constructs. In our opinion, anterolateral plating should only be used for specific fracture patterns such as a valgus displacement of the distal fracture fragment. In such cases, it would resist the deforming forces by buttressing against them. The posterior locking plate construct can be used safely in patients who are at a higher risk of developing wound complications as there is a limited soft tissue cover available on the medial and lateral sides.

There are several strengths of our study. This was a first-of-its-kind study comparing four different internal fixation modalities for low, extra-articular distal tibia fracture in a human cadaveric fracture model. We used a well-established and validated fracture model and biomechanical testing protocols. Furthermore, we standardized the bending loads used in the study by calculating the length of the distal fragment as a percentage of the total tibial length. Moreover, all the implants used for our study were procured from a single manufacturer. Finally, all cadaveric dissections, implant placement, and osteotomies were done uniformly with utmost care.

Nevertheless, there are certain limitations of our study. Foremost is the fact that we could not compare the bone mineral density of all the bones included in the study owing to restrictions imposed by the COVID-19 pandemic. Another limitation is that the fracture model selected for our study represents the “worst possible pattern” of an extra-articular fracture without considering the presence or absence of a concomitant fibula fracture. It is well known that the absence or stabilization of a fibular fracture confers additional stability to the tibial fracture, and distal tibial fractures with some bone contact and minimal comminution would inherently be more stable. Third, being a biomechanical study, this study did not take into account progressive fracture healing, which is usually observed in the clinical scenario; stability of the fracture implant construct can be expected to increase with time as the bone heals and this healing would add to the biomechanical stability. Individual patient parameters such as age, sex, and nutritional status also affect fracture healing to a large extent [6].

## Conclusions

The findings of our study conclude that, in cases where the locked plating technique is necessary, it is advisable to avoid the use of the anterolateral locking plate because of its inferior biomechanical stability in managing such fracture patterns. Despite the study's limitations, we have successfully illustrated that locked intramedullary nailing offers superior biomechanical stability for the internal fixation of low, extra-articular distal tibial fractures.
